# Supplementary material for: Examining influential factors in newly diagnosed cancer patients and survivors: Emphasizing distress, self-care ability, peer support, health perception, daily life activity, and the role of time since diagnosis
Source: PLoS One. 2023 Sep 1;18(9):e0291064. doi: 10.1371/journal.pone.0291064 (PMC10473484; doi:10.1371/journal.pone.0291064)
Supplement: S1 File — (DOCX) [file pone.0291064.s002.docx]

| Table 1. HTMT Ratio for discriminant validity | | | | | |
| --- | --- | --- | --- | --- | --- |
|  | **Original Est.** | **Bootstrap Mean** | **Bootstrap SD** | **T Stat.** | **CI**  **[5%, 95%]** |
| Peer support 🡪 Time since diagnosis | 0.012 | 0.033 | 0.019 | 0.613 | [0.005, 0.080] * |
| Peer support 🡪 Mental distress | 0.400 | 0.400 | 0.038 | 10.430 | [0.323, 0.474] * |
| Peer support 🡪 Difficulty of daily life activity | 0.199 | 0.199 | 0.045 | 4.400 | [0.111, 0.288] * |
| Peer support 🡪 Self-care | 0.350 | 0.350 | 0.033 | 10.685 | [0.284, 0.413] * |
| Peer support 🡪 Age | 0.095 | 0.096 | 0.033 | 2.885 | [0.031, 0.161] * |
| Peer support 🡪 Gender | 0.062 | 0.069 | 0.026 | 2.407 | [0.026, 0.126] * |
| Peer support 🡪 Education | 0.076 | 0.081 | 0.027 | 2.861 | [0.036, 0.138] * |
| Peer support 🡪 Health insurance | 0.034 | 0.046 | 0.028 | 1.199 | [0.007, 0.116] * |
| Peer support 🡪 Income | 0.171 | 0.171 | 0.034 | 4.990 | [0.104, 0.239] * |
| Peer support 🡪 Perception of health | 0.254 | 0.254 | 0.033 | 7.684 | [0.190, 0.319] * |
| Time since diagnosis 🡪 Mental distress | 0.065 | 0.067 | 0.028 | 2.308 | [0.021, 0.128] * |
| Time since diagnosis 🡪 The difficulty of daily life activity | 0.018 | 0.043 | 0.018 | 0.967 | [0.015, 0.086] * |
| Time since diagnosis 🡪 Self-care | 0.024 | 0.031 | 0.022 | 1.099 | [0.001, 0.081] * |
| Time since diagnosis 🡪 Age | 0.200 | 0.200 | 0.027 | 7.451 | [0.146, 0.252] * |
| Time since diagnosis 🡪 Gender | 0.072 | 0.072 | 0.029 | 2.530 | [0.016, 0.129] * |
| Time since diagnosis 🡪 Education | 0.040 | 0.042 | 0.025 | 1.585 | [0.002, 0.096] * |
| Time since diagnosis 🡪 Health insurance | 0.002 | 0.019 | 0.014 | 0.124 | [0.001, 0.052] * |
| Time since diagnosis 🡪 Income | 0.007 | 0.023 | 0.018 | 0.399 | [0.001, 0.065] * |
| Time since diagnosis 🡪 Perception of health | 0.057 | 0.057 | 0.028 | 1.989 | [0.006, 0.115] * |
| Mental distress 🡪 The difficulty of daily life activity | 0.411 | 0.411 | 0.042 | 9.722 | [0.328, 0.495] * |
| Mental distress 🡪 Self-care | 0.303 | 0.303 | 0.031 | 9.740 | [0.241, 0.363] * |
| Mental distress 🡪 Age | 0.070 | 0.072 | 0.030 | 2.297 | [0.021, 0.137] * |
| Mental distress 🡪 Gender | 0.086 | 0.087 | 0.029 | 2.971 | [0.033, 0.145] * |
| Mental distress 🡪 Education | 0.073 | 0.077 | 0.026 | 2.824 | [0.034, 0.132] * |
| Mental distress 🡪 Health insurance | 0.016 | 0.034 | 0.019 | 0.810 | [0.009, 0.084] * |
| Mental distress 🡪 Income | 0.110 | 0.110 | 0.032 | 3.465 | [0.049, 0.173] * |
| Mental distress 🡪 Perception of health | 0.318 | 0.318 | 0.031 | 10.151 | [0.256, 0.378] * |
| The difficulty of daily life activity 🡪 Self-care | 0.419 | 0.418 | 0.036 | 11.637 | [0.346, 0.487] * |
| The difficulty of daily life activity 🡪 Age | 0.193 | 0.196 | 0.030 | 6.376 | [0.138, 0.256] * |
| The difficulty of daily life activity 🡪 Gender | 0.059 | 0.068 | 0.025 | 2.375 | [0.027, 0.122] * |
| The difficulty of daily life activity 🡪 Education | 0.162 | 0.163 | 0.036 | 4.515 | [0.092, 0.233] * |
| The difficulty of daily life activity 🡪 Health insurance | 0.068 | 0.081 | 0.033 | 2.061 | [0.035, 0.162] * |
| The difficulty of daily life activity 🡪 Income | 0.298 | 0.298 | 0.037 | 8.002 | [0.224, 0.369] * |
| The difficulty of daily life activity 🡪 Perception of health | 0.511 | 0.511 | 0.029 | 17.802 | [0.454, 0.567] * |
| Self-care 🡪 Age | 0.094 | 0.094 | 0.028 | 3.401 | [0.040, 0.148] * |
| Self-care 🡪 Gender | 0.009 | 0.024 | 0.018 | 0.484 | [0.001, 0.066] * |
| Self-care 🡪 Education | 0.118 | 0.118 | 0.029 | 4.038 | [0.061, 0.175] * |
| Self-care 🡪 Health insurance | 0.031 | 0.032 | 0.018 | 1.708 | [0.002, 0.069] * |
| Self-care 🡪 Income | 0.133 | 0.133 | 0.030 | 4.443 | [0.074, 0.192] * |
| Self-care 🡪 Perception of health | 0.564 | 0.564 | 0.022 | 25.418 | [0.520, 0.606] * |
| Age 🡪 Gender | 0.140 | 0.141 | 0.029 | 4.798 | [0.083, 0.197] * |
| Age 🡪 Education | 0.055 | 0.055 | 0.027 | 2.061 | [0.005, 0.108] * |
| Age 🡪 Health insurance | 0.106 | 0.105 | 0.047 | 2.266 | [0.017, 0.198] * |
| Age 🡪 Income | 0.141 | 0.141 | 0.029 | 4.795 | [0.082, 0.197] * |
| Age 🡪 Perception of health | 0.038 | 0.040 | 0.024 | 1.604 | [0.002, 0.090] * |
| Gender 🡪 Education | 0.028 | 0.033 | 0.023 | 1.213 | [0.001, 0.084] * |
| Gender 🡪 Health insurance | 0.024 | 0.031 | 0.021 | 1.164 | [0.002, 0.077] * |
| Gender 🡪 Income | 0.074 | 0.074 | 0.028 | 2.658 | [0.019, 0.128] * |
| Gender 🡪 Perception of health | 0.082 | 0.082 | 0.028 | 2.882 | [0.026, 0.138] * |
| Education 🡪 Health insurance | 0.081 | 0.080 | 0.031 | 2.613 | [0.016, 0.139] * |
| Education 🡪 Income | 0.403 | 0.403 | 0.024 | 17.014 | [0.357, 0.450] * |
| Education 🡪 Perception of health | 0.205 | 0.205 | 0.027 | 7.525 | [0.151, 0.258] * |
| Health insurance 🡪 Income | 0.079 | 0.079 | 0.033 | 2.376 | [0.014, 0.146] * |
| Health insurance 🡪 Perception of health | 0.058 | 0.058 | 0.024 | 2.437 | [0.012, 0.106] * |
| Income 🡪 Perception of health | 0.249 | 0.249 | 0.029 | 8.653 | [0.192, 0.305] * |

Table 2. All moderation effects.

|  | **Original Est.** | **Bootstrap Mean** | **Bootstrap SD** | **T Stat.** | **CI**  **[5%, 95%]** |
| --- | --- | --- | --- | --- | --- |
| Peer support*Age🡪 Perception of health | 0.019 | 0.019 | 0.022 | 0.845 | [-0.024, 0.062] |
| Time since diagnosis*Age🡪 Perception of health | 0.011 | 0.011 | 0.023 | 0.485 | [-0.034, 0.056] |
| The difficulty of daily life activity*Age🡪 Perception of health | 0.057 | 0.057 | 0.024 | 2.339 | [0.007, 0.102] * |
| Self-care*Age🡪 Perception of health | -0.021 | -0.021 | 0.024 | -0.846 | [-0.070, 0.026] |
| Peer support*Gender🡪 Perception of health | -0.009 | -0.009 | 0.023 | -0.383 | [-0.054, 0.036] |
| Time since diagnosis*Gender🡪 Perception of health | 0.006 | 0.007 | 0.022 | 0.299 | [-0.035, 0.049] |
| The difficulty of daily life activity* Gender🡪 Perception of health | -0.010 | -0.010 | 0.028 | -0.358 | [-0.064, 0.046] |
| Self-care* Gender🡪 Perception of health | -0.047 | -0.046 | 0.026 | -1.800 | [-0.097, 0.005] |
| Peer support*Education🡪 Perception of health | -0.032 | -0.032 | 0.024 | -1.345 | [-0.079, 0.015] |
| Time since diagnosis* Education🡪 Perception of health | -0.038 | -0.038 | 0.022 | -1.733 | [-0.080, 0.006] |
| The difficulty of daily life activity* Education🡪 Perception of health | -0.043 | -0.043 | 0.024 | -1.729 | [-0.090, 0.006] |
| Self-care* Education🡪 Perception of health | 0.069 | 0.069 | 0.029 | 2.807 | [0.021, 0.117] * |
| Peer support*Income🡪 Perception of health | 0.012 | 0.011 | 0.024 | 0.482 | [-0.037, 0.058] |
| Time since diagnosis* Income🡪 Perception of health | -0.034 | -0.034 | 0.023 | -1.491 | [-0.080, 0.010] |
| The difficulty of daily life activity* Income🡪 Perception of health | -0.039 | -0.038 | 0.023 | -1.666 | [-0.084, 0.008] |
| Self-care* Income🡪 Perception of health | 0.052 | 0.052 | 0.026 | 2.012 | [0.001, 0.103] * |
| Peer support*Health insurance🡪 Perception of health | 0.035 | -0.038 | 0.591 | 0.060 | [-1.304, 0.383] |
| Time since diagnosis* Health insurance🡪 Perception of health | -0.047 | 0.078 | 0.857 | -0.055 | [-0.553, 2.265] |
| The difficulty of daily life activity* Health insurance🡪 Perception of health | 0.063 | 0.193 | 0.926 | 0.068 | [-0.745, 2.577] |
| Self-care* Health insurance | 0.029 | -0.248 | 1.156 | 0.025 | [-3.929, 0.651] |

Table 3. Effect sizes (f Square).

|  | Peer support | Time since diagnosis | Mental distress | The difficulty of daily life activity | Self-care | Age | Gender | Education | Health insurance | Income | Perception of health |
| --- | --- | --- | --- | --- | --- | --- | --- | --- | --- | --- | --- |
| Peer support | 0.000 | 0.000 | 0.110 | 0.000 | 0.054 | 0.000 | 0.000 | 0.000 | 0.000 | 0.000 | 0.001 |
| Time since diagnosis | 0.000 | 0.000 | 0.004 | 0.000 | 0.000 | 0.000 | 0.000 | 0.000 | 0.000 | 0.000 | 0.005 |
| Mental health | 0.000 | 0.000 | 0.000 | 0.118 | 0.018 | 0.000 | 0.000 | 0.000 | 0.000 | 0.000 | 0.000 |
| Daily life activity | 0.000 | 0.000 | 0.118 | 0.000 | 0.071 | 0.000 | 0.000 | 0.000 | 0.000 | 0.000 | 0.081 |
| Self-care | 0.054 | 0.000 | 0.018 | 0.071 | 0.000 | 0.000 | 0.000 | 0.000 | 0.000 | 0.000 | 0.279 |
| Age | 0.000 | 0.051 | 0.000 | 0.030 | 0.006 | 0.000 | 0.000 | 0.000 | 0.000 | 0.000 | 0.003 |
| Gender | 0.000 | 0.012 | 0.009 | 0.002 | 0.000 | 0.000 | 0.000 | 0.000 | 0.000 | 0.000 | 0.005 |
| Education | 0.000 | 0.002 | 0.001 | 0.001 | 0.004 | 0.000 | 0.000 | 0.000 | 0.000 | 0.000 | 0.010 |
| Health insurance | 0.000 | 0.001 | 0.000 | 0.001 | 0.002 | 0.000 | 0.000 | 0.000 | 0.000 | 0.000 | 0.008 |
| Income | 0.023 | 0.001 | 0.002 | 0.031 | 0.000 | 0.000 | 0.000 | 0.000 | 0.000 | 0.000 | 0.013 |
| Perception of health | 0.000 | 0.000 | 0.000 | 0.000 | 0.000 | 0.000 | 0.000 | 0.000 | 0.000 | 0.000 | 0.000 |

Table 4. Indirect and total paths.

| **Path coefficients** | **Original Est.** | **SD** | **T Stat.** | **CI**  **[5%, 95%]** |
| --- | --- | --- | --- | --- |
| **Indirect effects of control variables** |  |  |  |  |
| Age 🡪Peer support🡪Mental distress | -0.037 | 0.009 | -3.891 | [-0.056, -0.019] * |
| Age🡪Peer support🡪Mental distress 🡪 The difficulty of daily life activity | -0.012 | 0.003 | -3.457 | [-0.019, -0.006] * |
| Age🡪Peer support🡪Mental distress 🡪 The difficulty of daily life activity 🡪Self-care | 0.003 | 0.001 | 3.158 | [0.001, 0.005] * |
| Age🡪Peer support🡪Mental distress 🡪 The difficulty of daily life activity 🡪Self-care 🡪Perception of health | 0.001 | 0.000 | 3.098 | [0.001, 0.002] * |
| Age 🡪Time since diagnosis🡪Mental distress | -0.013 | 0.007 | -1.899 | [-0.027, 0.000] |
| Age 🡪Time since diagnosis🡪Mental distress 🡪 The difficulty of daily life activity | -0.004 | 0.002 | -1.807 | [-0.009, -0.000] * |
| Age 🡪Time since diagnosis🡪Mental distress 🡪 The difficulty of daily life activity 🡪Self-care | 0.001 | 0.001 | 1.728 | [0.000, 0.002] * |
| Age 🡪Time since diagnosis🡪Mental distress 🡪 The difficulty of daily life activity 🡪Self-care🡪Perception of health | 0.000 | 0.000 | 1.721 | [0.000, 0.001] * |
| Age 🡪Mental distress🡪 The difficulty of daily life activity | -0.007 | 0.010 | -0.641 | [-0.027, 0.013] |
| Age 🡪Mental distress🡪 The difficulty of daily life activity 🡪Self-care | 0.002 | 0.003 | 0.636 | [-0.004, 0.007] |
| Age 🡪Mental distress🡪 The difficulty of daily life activity 🡪Self-care🡪Perception of health | 0.001 | 0.001 | 0.634 | [-0.002, 0.003] |
| Age 🡪 The difficulty of daily life activity 🡪Self-care | -0.043 | 0.010 | -4.334 | [-0.065, -0.020] * |
| Age 🡪 The difficulty of daily life activity 🡪Self-care🡪Perception of health | -0.020 | 0.005 | -4.055 | [-0.030, -0.011] * |
| Age 🡪Self-care🡪Perception of health | -0.032 | 0.012 | -2.641 | [-0.056, -0.009] * |
| Gender 🡪Peer support🡪Mental distress | -0.025 | 0.009 | -2.746 | [-0.044, -0.007] * |
| Gender 🡪Peer support🡪Mental distress 🡪 The difficulty of daily life activity | -0.008 | 0.003 | -2.527 | [-0.015, -0.002] * |
| Gender 🡪Peer support🡪Mental distress 🡪 The difficulty of daily life activity 🡪Self-care | 0.002 | 0.001 | 2.403 | [0.001, 0.004] * |
| Gender 🡪Peer support🡪Mental distress 🡪 The difficulty of daily life activity 🡪Self-care 🡪Perception of health | 0.001 | 0.000 | 2.354 | [0.000, 0.002] * |
| Gender 🡪Time since diagnosis🡪Mental distress | -0.006 | 0.004 | -1.683 | [-0.015, -0.000] * |
| Gender 🡪Time since diagnosis🡪Mental distress 🡪 The difficulty of daily life activity | -0.002 | 0.001 | -1.597 | [-0.005, -0.000] * |
| Gender 🡪Time since diagnosis🡪Mental distress 🡪 The difficulty of daily life activity 🡪Self-care | 0.001 | 0.000 | 1.544 | [0.000, 0.001] * |
| Gender 🡪Time since diagnosis🡪Mental distress 🡪 The difficulty of daily life activity 🡪Self-care🡪Perception of health | 0.000 | 0.000 | 1.537 | [0.000, 0.001] * |
| Gender 🡪Mental distress🡪 The difficulty of daily life activity | 0.029 | 0.010 | 3.007 | [0.011, 0.049] * |
| Gender 🡪Mental distress🡪 The difficulty of daily life activity 🡪Self-care | -0.008 | 0.003 | -2.796 | [-0.013, -0.003] * |
| Gender 🡪Mental distress🡪 The difficulty of daily life activity 🡪Self-care🡪Perception of health | -0.003 | 0.001 | -2.756 | [-0.006, -0.001] * |
| Gender 🡪 The difficulty of daily life activity 🡪Self-care | -0.009 | 0.007 | -1.302 | [-0.023, 0.005] |
| Gender 🡪 The difficulty of daily life activity 🡪Self-care🡪Perception of health | -0.004 | 0.003 | -1.295 | [-0.011, 0.002] |
| Gender 🡪Self-care🡪Perception of health | -0.002 | 0.012 | -0.178 | [-0.025, 0.020] |
| Education 🡪Peer support🡪Mental distress | -0.003 | 0.010 | -0.277 | [-0.022, 0.017] |
| Education 🡪Peer support🡪Mental distress 🡪 The difficulty of daily life activity | -0.001 | 0.003 | -0.275 | [-0.007, 0.005] |
| Education 🡪Peer support🡪Mental distress 🡪 The difficulty of daily life activity 🡪Self-care | 0.000 | 0.001 | 0.273 | [-0.001, 0.002] |
| Education 🡪Peer support🡪Mental distress 🡪 The difficulty of daily life activity 🡪Self-care 🡪Perception of health | 0.000 | 0.000 | 0.272 | [-0.001, 0.001] |
| Education 🡪Time since diagnosis🡪Mental distress | -0.003 | 0.002 | -1.146 | [-0.008, 0.001] |
| Education 🡪Time since diagnosis🡪Mental distress 🡪 The difficulty of daily life activity | -0.001 | 0.001 | -1.115 | [-0.003, 0.000] |
| Education 🡪Time since diagnosis🡪Mental distress 🡪 The difficulty of daily life activity 🡪Self-care | 0.000 | 0.000 | 1.088 | [-0.000, 0.001] |
| Education 🡪Time since diagnosis🡪Mental distress 🡪 The difficulty of daily life activity 🡪Self-care🡪Perception of health | 0.000 | 0.000 | 1.085 | [-0.000, 0.000] |
| Education 🡪Mental distress🡪 The difficulty of daily life activity | -0.009 | 0.010 | -0.832 | [-0.030, 0.011] |
| Education 🡪Mental distress🡪 The difficulty of daily life activity 🡪Self-care | 0.002 | 0.003 | 0.812 | [-0.003, 0.008] |
| Education 🡪Mental distress🡪 The difficulty of daily life activity 🡪Self-care🡪Perception of health | 0.001 | 0.001 | 0.810 | [-0.001, 0.004] |
| Education 🡪 The difficulty of daily life activity 🡪Self-care | 0.008 | 0.008 | 0.992 | [-0.008, 0.024] |
| Education 🡪 The difficulty of daily life activity 🡪Self-care🡪Perception of health | 0.004 | 0.004 | 0.985 | [-0.004, 0.011] |
| Education 🡪Self-care🡪Perception of health | 0.028 | 0.013 | 2.156 | [0.002, 0.054] * |
| Income 🡪Peer support🡪Mental distress | -0.053 | 0.012 | -4.422 | [-0.077, -0.031] * |
| Income 🡪Peer support🡪Mental distress 🡪 The difficulty of daily life activity | -0.017 | 0.004 | -3.932 | [-0.026, -0.009] * |
| Income 🡪Peer support🡪Mental distress 🡪 The difficulty of daily life activity 🡪Self-care | 0.004 | 0.001 | 3.665 | [0.002, 0.007] * |
| Income 🡪Peer support🡪Mental distress 🡪 The difficulty of daily life activity 🡪Self-care 🡪Perception of health | 0.002 | 0.001 | 3.569 | [0.001, 0.003] * |
| Income 🡪Time since diagnosis🡪Mental distress | -0.002 | 0.002 | -0.756 | [-0.008, 0.002] |
| Income 🡪Time since diagnosis🡪Mental distress 🡪 The difficulty of daily life activity | -0.001 | 0.001 | -0.737 | [-0.002, 0.000] |
| Income 🡪Time since diagnosis🡪Mental distress 🡪 The difficulty of daily life activity 🡪Self-care | 0.000 | 0.000 | 0.725 | [-0.000, 0.001] |
| Income 🡪Time since diagnosis🡪Mental distress 🡪 The difficulty of daily life activity 🡪Self-care🡪Perception of health | 0.000 | 0.000 | 0.725 | [-0.000, 0.000] |
| Income 🡪Mental distress🡪 The difficulty of daily life activity | -0.014 | 0.011 | -1.273 | [-0.036, 0.008] |
| Income 🡪Mental distress🡪 The difficulty of daily life activity 🡪Self-care | 0.004 | 0.003 | 1.251 | [-0.002, 0.009] |
| Income 🡪Mental distress🡪 The difficulty of daily life activity 🡪Self-care🡪Perception of health | 0.002 | 0.001 | 1.241 | [-0.001, 0.004] |
| Income 🡪 The difficulty of daily life activity 🡪Self-care | 0.047 | 0.012 | 4.100 | [0.026, 0.071] * |
| Income 🡪 The difficulty of daily life activity 🡪Self-care🡪Perception of health | 0.021 | 0.005 | 3.904 | [0.012, 0.033] * |
| Income 🡪Self-care🡪Perception of health | -0.005 | 0.014 | -0.382 | [-0.033, 0.022] |
| Health insurance 🡪Peer support🡪Mental distress | 0.000 | 0.011 | 0.022 | [-0.021, 0.023] |
| Health insurance 🡪Peer support🡪Mental distress 🡪 The difficulty of daily life activity | 0.000 | 0.004 | 0.022 | [-0.007, 0.008] |
| Health insurance 🡪Peer support🡪Mental distress 🡪 The difficulty of daily life activity 🡪Self-care | -0.000 | 0.001 | -0.022 | [-0.002, 0.002] |
| Health insurance 🡪Peer support🡪Mental distress 🡪 The difficulty of daily life activity 🡪Self-care 🡪Perception of health | -0.000 | 0.000 | -0.021 | [-0.001, 0.001] |
| Health insurance 🡪Time since diagnosis🡪Mental distress | -0.002 | 0.002 | -1.111 | [-0.006, 0.001] |
| Health insurance 🡪Time since diagnosis🡪Mental distress 🡪 The difficulty of daily life activity | -0.001 | 0.001 | -1.081 | [-0.002, 0.000] |
| Health insurance 🡪Time since diagnosis🡪Mental distress 🡪 The difficulty of daily life activity 🡪Self-care | 0.000 | 0.000 | 1.052 | [-0.000, 0.000] |
| Health insurance 🡪Time since diagnosis🡪Mental distress 🡪 Difficulty of daily life activity 🡪Self-care🡪Perception of health | 0.000 | 0.000 | 1.050 | [-0.000, 0.000] |
| Health insurance 🡪Mental distress🡪 The difficulty of daily life activity | 0.000 | 0.011 | 0.040 | [-0.019, 0.023] |
| Health insurance 🡪Mental distress🡪 The difficulty of daily life activity 🡪Self-care | -0.000 | 0.003 | -0.039 | [-0.006, 0.005] |
| Health insurance 🡪Mental distress🡪 The difficulty of daily life activity 🡪Self-care🡪Perception of health | -0.000 | 0.001 | -0.039 | [-0.003, 0.002] |
| Health insurance 🡪 Difficulty of daily life activity 🡪Self-care | -0.006 | 0.007 | -0.783 | [-0.022, 0.006] |
| Health insurance 🡪 Difficulty of daily life activity 🡪Self-care🡪Perception of health | -0.003 | 0.003 | -0.775 | [-0.010, 0.003] |
| Health insurance 🡪Self-care🡪Perception of health | 0.019 | 0.008 | 2.366 | [0.003, 0.035] * |
| **Total direct effects of study variables** |  |  |  |  |
| Peer support 🡪 Mental distress | -0.318 | 0.031 | -10.131 | [-0.379, -0.256] * |
| Peer support 🡪 Difficulty of daily life activity | -0.100 | 0.016 | -6.242 | [-0.135, -0.071] * |
| Peer support 🡪 Self-care | 0.291 | 0.028 | 10.424 | [0.238, 0.347] * |
| Peer support 🡪 Perception of health | 0.186 | 0.026 | 7.078 | [0.134, 0.237] * |
| Time since diagnosis 🡪 Mental distress | -0.058 | 0.029 | -2.011 | [-0.114, -0.002] * |
| Time since diagnosis 🡪 The difficulty of daily life activity | -0.018 | 0.010 | -1.900 | [-0.038, -0.001] * |
| Time since diagnosis 🡪 Self-care | 0.013 | 0.007 | 1.908 | [0.001, 0.026] * |
| Time since diagnosis 🡪 Perception of health | 0.066 | 0.024 | 2.712 | [0.018, 0.113] * |
| Mental distress 🡪 The difficulty of daily life activity | 0.316 | 0.036 | 8.856 | [0.246, 0.387] * |
| Mental distress 🡪 Self-care | -0.216 | 0.030 | -7.322 | [-0.274, -0.157] * |
| Mental distress 🡪 Perception of health | -0.174 | 0.020 | -8.477 | [-0.215, -0.134] * |
| The difficulty of daily life activity 🡪 Self-care | -0.262 | 0.035 | -7.414 | [-0.331, -0.192] * |
| The difficulty of daily life activity 🡪 Perception of health | -0.359 | 0.028 | -12.790 | [-0.414, -0.303] * |
| Self-care 🡪 Perception of health | 0.450 | 0.026 | 17.570 | [0.399, 0.499] * |
| **Total direct effects of control variables** |  |  |  |  |
| Age 🡪 Peer support | 0.116 | 0.028 | 4.087 | [0.062, 0.173] * |
| Age 🡪 Time since diagnosis | 0.225 | 0.028 | 7.954 | [0.171, 0.281] * |
| Age 🡪 Mental distress | -0.071 | 0.033 | -2.154 | [-0.138, -0.008] * |
| Age 🡪 The difficulty of daily life activity | 0.143 | 0.029 | 4.922 | [0.084, 0.199] * |
| Age 🡪 Self-care | -0.074 | 0.028 | -2.599 | [-0.130, -0.017] * |
| Age 🡪 Perception of health | -0.003 | 0.027 | -0.118 | [-0.055, 0.048] |
| Gender 🡪 Peer support | 0.080 | 0.028 | 2.890 | [0.024, 0.133] * |
| Gender 🡪 Time since diagnosis | 0.108 | 0.029 | 3.744 | [0.052, 0.165] * |
| Gender 🡪 Mental distress | 0.060 | 0.029 | 2.108 | [0.005, 0.117] * |
| Gender 🡪 The difficulty of daily life activity | 0.054 | 0.027 | 1.972 | [0.000, 0.108] * |
| Gender 🡪 Self-care | -0.009 | 0.028 | -0.317 | [-0.066, 0.046] |
| Gender 🡪 Perception of health | -0.062 | 0.028 | -2.244 | [-0.117, -0.008] * |
| Education 🡪 Peer support | 0.009 | 0.031 | 0.280 | [-0.053, 0.070] |
| Education 🡪 Time since diagnosis | 0.046 | 0.031 | 1.489 | [-0.016, 0.106] |
| Education 🡪 Mental distress | -0.033 | 0.033 | -0.975 | [-0.098, 0.033] |
| Education 🡪 The difficulty of daily life activity | -0.041 | 0.032 | -1.283 | [-0.103, 0.022] |
| Education 🡪 Self-care | 0.079 | 0.033 | 2.409 | [0.014, 0.143] * |
| Education 🡪 Perception of health | 0.130 | 0.030 | 4.349 | [0.071, 0.188] * |
| Health insurance 🡪 Peer support | -0.001 | 0.036 | -0.022 | [-0.074, 0.066] |
| Health insurance 🡪 Time since diagnosis | 0.031 | 0.021 | 1.502 | [-0.011, 0.071] |
| Health insurance 🡪 Mental distress | -0.000 | 0.034 | -0.006 | [-0.061, 0.072] |
| Health insurance 🡪 The difficulty of daily life activity | 0.022 | 0.030 | 0.731 | [-0.023, 0.090] |
| Health insurance 🡪 Self-care | 0.037 | 0.021 | 1.778 | [-0.002, 0.079] |
| Health insurance 🡪 Perception of health | 0.083 | 0.031 | 2.707 | [0.023, 0.144] * |
| Income 🡪 Peer support | 0.165 | 0.033 | 5.084 | [0.103, 0.228] * |
| Income 🡪 Time since diagnosis | 0.031 | 0.031 | 0.985 | [-0.030, 0.093] |
| Income 🡪 Mental distress | -0.098 | 0.036 | -2.717 | [-0.169, -0.028] * |
| Income 🡪 The difficulty of daily life activity | -0.212 | 0.035 | -5.989 | [-0.280, -0.141] * |
| Income 🡪 Self-care | 0.093 | 0.035 | 2.694 | [0.026, 0.162] * |
| Income 🡪 Perception of health | 0.198 | 0.031 | 6.334 | [0.138, 0.260] * |
