# Supplementary material for: Examining influential factors in newly diagnosed cancer patients and survivors: Emphasizing distress, self-care ability, peer support, health perception, daily life activity, and the role of time since diagnosis
Source: PLoS One. 2023 Sep 1;18(9):e0291064. doi: 10.1371/journal.pone.0291064 (PMC10473484; doi:10.1371/journal.pone.0291064)
Supplement: S2 File — (DOCX) [file pone.0291064.s003.docx]

Table 1. Multi-group analysis comparing cancer survivors with patients undergoing cancer treatment.

|  | **P value** |
| --- | --- |
| Peer support 🡪 Mental distress | 0.344 |
| Peer support 🡪 Self-care | 0.313 |
| Peer support 🡪 Perception of health | 0.052 |
| Time since diagnosis 🡪 Mental distress | 0.527 |
| Time since diagnosis 🡪 Perception of health | 0.319 |
| Mental distress 🡪 The difficulty of daily life activity | 0.642 |
| Mental distress 🡪 Self-care | 0.823 |
| The difficulty of daily life activity 🡪 Self-care | 0.614 |
| The difficulty of daily life activity 🡪 Perception of health | 0.403 |
| Self-care 🡪 Perception of health | 0.807 |
| Age 🡪 Peer support | 0.594 |
| Age 🡪 Time since diagnosis | 0.826 |
| Age 🡪 Mental distress | 0.722 |
| Age 🡪 The difficulty of daily life activity | 0.589 |
| Age 🡪 Self-care | 0.888 |
| Age 🡪 Perception of health | 0.152 |
| Gender 🡪 Peer support | 0.766 |
| Gender 🡪 Time since diagnosis | 0.753 |
| Gender 🡪 Mental distress | 0.883 |
| Gender 🡪 The difficulty of daily life activity | 0.417 |
| Gender 🡪 Self-care | 0.538 |
| Gender 🡪 Perception of health | 0.052 |
| Education 🡪 Peer support | 0.582 |
| Education 🡪 Time since diagnosis | 0.194 |
| Education 🡪 Mental distress | 0.824 |
| Education 🡪 The difficulty of daily life activity | 0.482 |
| Education 🡪 Self-care | 0.360 |
| Education 🡪 Perception of health | 0.387 |
| Income 🡪 Peer support | 0.620 |
| Income 🡪 Time since diagnosis | 0.185 |
| Income 🡪 Mental distress | 0.694 |
| Income 🡪 The difficulty of daily life activity | 0.613 |
| Income 🡪 Self-care | 0.154 |
| Income 🡪 Perception of health | 0.213 |
