# Supplementary material for: Examining influential factors in newly diagnosed cancer patients and survivors: Emphasizing distress, self-care ability, peer support, health perception, daily life activity, and the role of time since diagnosis
Source: PLoS One. 2023 Sep 1;18(9):e0291064. doi: 10.1371/journal.pone.0291064 (PMC10473484; doi:10.1371/journal.pone.0291064)
Supplement: Attachment — Submitted filename: Response to Reviewers.docx [file pone.0291064.s004.docx]

5. Review Comments to the Author

Reviewer #1: this paper reads well and the results are rather interesting.

in line 242 you say that '264 were slightly worried about getting reinfected with cancer'. I don't think cancer is widely recognised as an infectious disease and another word should be used instead of 'reinfected'.

**Response: We have rephrased the sentence to *“264 were slightly worried about experiencing a recurrence of cancer”***

In table 1 the sample size (N) should be added in the title of the table and the 'n' of each group should also be included in the title's row.

**Response: We have added the sample size to the table caption and group titles in each column.**
